# Reviewing umbrella reviews of systematic reviews of original studies on the effects of air pollution on disease

**DOI:** 10.1097/EE9.0000000000000324

**Published:** 2024-08-06

**Authors:** Bert Brunekreef, Kurt Straif, Neil Pearce

**Affiliations:** a Institute for Risk Assessment Sciences, Utrecht University, The Netherlands; b ISGlobal, Barcelona, Spain; c Boston College, Chestnut Hill, Massachusetts, USA; d London School of Hygiene and Tropical Medicine, England, UK

**What this study adds**

This commentary adds to the growing literature on the synthesis of environmental epidemiology studies. The commentary focuses on how to deal with reviews of systematic reviews of original studies on associations between long-term air pollution exposure and chronic disease outcomes. Reviewing such umbrella reviews poses special challenges that are not easily met. Our commentary provides some suggestions on how to move forward in reviewing evidence from studies in environmental epidemiology.

## Commentary

Does air pollution make you sick? To answer that question, a study was initiated some 50 years ago.^1^ That study showed that yes, air pollution makes you sick, and makes you die a few years earlier than folks living in clean air. This being an epidemiological study, it showed an *association* between air pollution and disease, which is not necessarily the same as *causation*. To support causation, one important requirement is to show that results are reproducible in another population (among other considerations), see Hill^2^ This was achieved in the American Cancer Society study.^3^ Because these findings potentially had large policy implications, an unprecedented independent reanalysis was conducted^4^ that corroborated the findings.

The results of these two studies were at the heart of the United States Environmental Protection Agency 1997 PM2.5 (airborne particles with an aerodynamic diameter of less than 2.5 micrometer) National Ambient Air Quality Standard, and of the WHO 2005 Air Quality Guidelines for PM2.5.

As the consequences of these findings for public health and environmental policy were large, the number of studies looking into the health effects of air pollution has exploded. In 1995, PUBMED listed 383 articles in response to “air pollution health.” In 2000, there were 683; in 2005, there were 978. In 2010, 1420. In 2015, 2496. In 2020, 4489 and in 2022, the latest year with full coverage, 5370. Some of these answered genuine new research questions, such as which components and sources were especially to blame, which populations were especially vulnerable, which biological mechanisms were responsible, etc. However, many studies were just repetitions with similar study designs in similar populations that did not contribute much to our understanding of the main research questions.

The ongoing tsunami of new studies presents significant challenges to reviewing the evidence. In the last 25 years or so, systematic reviews have become the dominant form of evidence synthesis in biomedical science, often combined with a quantitative meta-analysis of the associations between exposure and disease. “Systematic” suggests objectivity and reproducibility of the reviews, and while that surely is the intention, there are many systems around and little hard evidence that the results are truly “objective” and “reproducible.” As one example, the reviews to support the development of the latest WHO Air Quality Guidelines needed significant adaptation of the WHO Handbook for Guideline Development (2014 edition) to make it halfway suitable for evaluating the evidence on associations between air pollution and disease and mortality.^5^

The emphasis in most systematic review protocols is on being as inclusive as possible in the initial search. This typically leads to thousands of references unearthed by the initial search, which then need to be reduced to a small number of relevant and suitable studies. While the reasons for inclusion and exclusion are typically provided, the sheer volume of initial references makes it hard to avoid the possibility that some relevant articles are excluded by accident, even when the excluding is done in duplicate. Three systematic reviews on nitrogen dioxide and mortality were published recently within 1 year, but the overlap in studies included in the final analyses was not as large as expected.^6–8^ Two of the three used exactly the same risk-of-bias tool to evaluate risk-of-bias in individual studies, but the agreement of the results was poor.

Journals nowadays may require proof of prior registration of review protocols, preferably in the PROSPERO (international prospective register of systematic reviews) registry https://www.crd.york.ac.uk/prospero/. While this allows checking for duplicates, and basic review procedures, the PROSPERO center does not provide peer review and has recently resorted to basic automated checks of submitted protocols due to the very high number of protocols submitted for registration. Interestingly, 71% of the systematic reviews analyzed in this manuscript were not registered in PROSPERO.

On the contrary, there is a tsunami of systematic reviews in the biomedical literature. Whereas these were almost absent from the literature in the previous century, the number of “systematic reviews” in PUBMED has ballooned from 379 in 1999 to 52,184 in 2022. In the air pollution field, there were 226 systematic reviews listed in PUBMED in 2022 alone, while they were virtually absent before the year 2000. Many of these reviews were investigator-initiated and were not part of some effort of guideline or regulatory standard development. Reviews tend to be cited more often than research articles (which is what authors like), and because of that, journals are tempted to publish reviews, as this may boost their citation score (which is what editors and publishers like).

We have now reached the stage where we need to make sense of the amassed systematic reviews. The term “umbrella review” has been coined for this. These also were virtually absent in the previous century but are increasing as fast as the systematic reviews themselves, growing from 8 in 1999 to 749 in PUBMED in 2022.

One might question whether there is a place for umbrella reviews and whether they add important knowledge. One might argue that it is better to simply perform the best possible updated systematic review, making use of all of the previously published ones for the identification of eligible original studies and perhaps data extraction, rather than taking the umbrella review approach. This would involve quality assessment of the original studies for use in sensitivity analyses to explore reasons for heterogeneity (including a triangulation approach). One might argue that this will be more productive than an umbrella review approach which explores reasons for differences between the various systematic reviews (much of which will arise from the studies included or excluded and different, but often nonsatisfactory assessments of the quality of the original studies). However, such an updated systematic review would raise methodological issues that are beyond the scope of this commentary. Moreover, for better or worse, umbrella reviews are already happening, so it is useful to explore the methodological issues involved in this particular approach.

The current issue of Environmental Epidemiology has an article authored by Forastiere et al^9^, “Choices of morbidity outcomes and concentration-response functions for health risk assessment of long-term exposure to air pollution.”^9^ It proposes some 20 concentration-response functions for the effects of long-term exposure to common air pollutants on the incidence of a series of chronic disease endpoints. The approach taken by the authors was not to perform entirely new systematic reviews and meta-analyses of the vast literature, but rather to probe existing systematic reviews. Exposure-disease combinations were chosen largely based on recent Integrated Science Assessments from the United States Environmental Protection Agency. These assessments make careful analyses of the literature, including laboratory studies, to arrive at verdicts of associations being “causal,” “likely causal,” “suggestive of, but not sufficient to infer, a causal relationship,” “inadequate to infer a causal relationship”, and “not likely to be a causal relationship.”^10^ Only associations falling in the first two categories were further assessed.

Then, the authors searched for and evaluated published systematic reviews and meta-analyses. Following a detailed search string, they identified 75 different systematic reviews with meta-analyses for these 20 combinations. They then developed a set of criteria to judge the adequacy of these reviews to answer the question. These criteria were loosely based on the AMSTAR 2 (a critical appraisal tool for systematic reviews that include randomized or non-randomised studies of healthcare interventions) checklist^11^ to which they made a number of changes and additions to make them more suitable for judging systematic reviews on air pollution and incidence of chronic disease. Initially, they also developed an AMSTAR 2-like ranking of the quality of the evidence provided by the systematic reviews, but in consultation with us, they refrained from keeping that in the revised manuscript. There was a detailed exchange of viewpoints, the bottom line of which was that few of the original and adapted criteria can be used as “hard” evidence to prefer one systematic review over the other. The authors have now followed our suggestion to use the published systematic reviews as a source of effect estimates from original studies that can then be meta-analyzed independently for two of the investigated pollutant-outcome pairs. This approach gave reassuring results, that is, the joint effect estimate did not deviate much from the effect estimates in the separate systematic reviews. However, this may be different for other pollutant-outcome pairs. The authors therefore reported all effect estimates from the systematic reviews in e-appendix 2, Table A1.^9^ As is evident from this table, the effect estimates were similar for some pollutant-outcome pairs but not for others (notably PM2.5 and asthma in children, stroke, arterial fibrillation, and autism spectrum disorders). Readers may therefore hesitate to use just one of the published meta-analytic effect estimates for burden or impact assessments.

It is important to realize that all this rests on the identification and analysis of reviews rather than the original studies. The original studies may be fine, but the systematic review may not do a good job of summarizing the evidence. Or, there may be significant problems with some of the original studies that are not picked up by a systematic review. There is no way a review of systematic reviews can provide a definite solution to such problems. Of course, this is an even greater problem when reviewing reviews of systematic reviews. We freely admit that we are unable to judge whether the hundreds of original studies that made it into the 75 systematic reviews were adequately represented in either the systematic reviews themselves or in the reviews of these systematic reviews, which form the core of this comprehensive and courageous report.

It is interesting to note that others have evaluated systematic reviews of environmental health studies (notably on air pollution and reproductive and child health) from a methodological point of view.^12^ After examining no less than 177 systematic reviews, the authors found that only 18 of these used some kind of evidence grading system. A wide variety of approaches and systems was identified, effectively making it impossible to draw firm conclusions about these methods’ validity and results. The authors concluded that “Establishing the wider use of more appropriate evidence grading methods is instrumental both for strengthening systematic review methodologies, and for the effective development and implementation of environmental public health policies, particularly for protecting pregnant persons and children.” The key word here is “appropriate”—appropriate for evaluating the evidence from observational epidemiology that forms the scientific bedrock of environmental public health policies.

Another research team has focused on the reproducibility of systematic review search strategies in a random sample of 100 published systematic reviews.^13^ The authors concluded that out of 467 included database searches, “only 47 (10.4%) could be reproduced within 10% of the number of results from the original search; six searches differed by more than 1,000% between the originally reported number of results and the reproduction. Only one systematic review article provided the necessary search details to be fully reproducible.”

With such examples in mind, one must view the results of any attempt to draw firm conclusions from published systematic reviews with great reservation.

Is there a sensible way forward other than continuing to be overwhelmed by ever-increasing numbers of original studies, systematic reviews, and now umbrella reviews? We do not have the answers, but we think the following considerations could be useful.

When considering individual studies, there is a need to put more effort into the identification of key informative studies addressing questions of substance (e.g. the possibility of a particular type of bias). Moreover, instead of reducing studies to a single number (the relative risk with its confidence interval) and a crude classification of the “risk-of-bias,” we suggest to focus on what scientific questions we are trying to answer and which studies are most relevant (irrespective of their overall “risk-of-bias”). For a particular question, rather than simply scoring studies, it is important to identify the most likely sources of bias,^14^ and assess these using all of the relevant studies (no matter what their overall “risk of bias” may be). For example, if confounding by a particular factor (e.g. tobacco smoking) is of concern, what studies can best be used to assess this, and how do their results and conclusions compare? Which studies have the best data on such confounders, and what happens to the main effect estimates after confounder adjustment? Related to this, we support a triangulation approach^15^ in which studies that are likely to have biases in different directions (or the same bias to a greater or lesser extent) are compared.

Similar considerations apply when considering and contrasting systematic reviews of individual studies, that is, why do they give different results, how have they differed in terms of their inclusion and exclusion criteria, and the way that they have assessed possible biases? Once again, the focus should be on identifying and assessing the most likely sources of bias, rather than a mechanical scoring or “tick box” approach.

In both of these contexts, that is, reviewing individual studies and reviewing systematic reviews, a major challenge, obviously, is how to tell “key” informative studies from “nonkey” studies, and how to identify the most likely sources of bias. However, that challenge could be faced in consultation with subject matter and review methodology experts. Elements would include the quality of exposure assessment methods and results and other design issues that go beyond what is now addressed in “risk of bias” tools. Otherwise, there is a danger that the inadequacies of the algorithmic approach to reviewing individual studies will simply be reproduced at a higher level when reviewing systematic reviews. We consider that Forastiere et al^9^ have largely avoided these problems in their major and comprehensive umbrella review, but more generally this particular approach nevertheless perhaps raises as many methodological issues as it answers.

## Conflicts of interest

The authors declare that they have no conflicts of interest with regard to the content of this report.
